# Characteristics and course of patients with AA amyloidosis: single centre experience with 174 patients from Turkey

**DOI:** 10.1093/rheumatology/kead465

**Published:** 2023-09-20

**Authors:** Murat Bektas, Nevzat Koca, Emin Oguz, Selma Sari, Gizem Dagci, Burak Ince, Pelin Karaca Ozer, Besim Fazil Agargun, Yasemin Yalcinkaya, Bahar Artim-Esen, Lale Ocal, Murat Inanc, Ahmet Gul

**Affiliations:** Division of Rheumatology, Department of Internal Medicine, Istanbul Faculty of Medicine, Istanbul University, Fatih, Istanbul, Turkey; Division of Rheumatology, Department of Internal Medicine, Istanbul Faculty of Medicine, Istanbul University, Fatih, Istanbul, Turkey; Division of Rheumatology, Department of Internal Medicine, Istanbul Faculty of Medicine, Istanbul University, Fatih, Istanbul, Turkey; Division of Rheumatology, Department of Internal Medicine, Istanbul Faculty of Medicine, Istanbul University, Fatih, Istanbul, Turkey; Department of Internal Medicine, Istanbul Faculty of Medicine, Istanbul University, Fatih, Istanbul, Turkey; Division of Rheumatology, Department of Internal Medicine, Istanbul Faculty of Medicine, Istanbul University, Fatih, Istanbul, Turkey; Department of Cardiology, Istanbul Faculty of Medicine, Istanbul University, Fatih, Istanbul, Turkey; Department of Internal Medicine, Istanbul Faculty of Medicine, Istanbul University, Fatih, Istanbul, Turkey; Division of Rheumatology, Department of Internal Medicine, Istanbul Faculty of Medicine, Istanbul University, Fatih, Istanbul, Turkey; Division of Rheumatology, Department of Internal Medicine, Istanbul Faculty of Medicine, Istanbul University, Fatih, Istanbul, Turkey; Division of Rheumatology, Department of Internal Medicine, Istanbul Faculty of Medicine, Istanbul University, Fatih, Istanbul, Turkey; Division of Rheumatology, Department of Internal Medicine, Istanbul Faculty of Medicine, Istanbul University, Fatih, Istanbul, Turkey; Division of Rheumatology, Department of Internal Medicine, Istanbul Faculty of Medicine, Istanbul University, Fatih, Istanbul, Turkey

**Keywords:** AA amyloidosis, FMF, *MEFV*, IL-1 inhibitors, amyloid storm

## Abstract

**Objectives:**

This study aimed to evaluate the clinical, laboratory and genetic characteristics and outcomes of patients with AA amyloidosis.

**Methods:**

Patients followed up in a tertiary referral centre in Turkey with the diagnosis of inflammatory rheumatic diseases and immunohistologically proven AA amyloidosis were included in the study and retrospectively analysed.

**Results:**

Among 184 patients with the diagnosis of AA amyloidosis, 174 (83 female, 91 male) were included in the analysis. The most common cause of AA amyloidosis was FMF (78.7%), and 91% of FMF-AA amyloidosis patients were carrying the p.M694V variant (74.1% homozygous). AA amyloidosis was identified earlier in patients with homozygous or compound heterozygous *MEFV* exon 10 variants compared with the heterozygous patients (27, 30 and 41 years, respectively). Patients with an estimated glomerular filtration rate <60 ml/min at admission had a higher frequency of progression to end-stage renal disease (*P* < 0.001). The overall mortality rate was 15.3% and it increased gradually in association with the amyloid burden (10% in patients with renal, 15% in renal + gastrointestinal and 43% in those with additional cardiac involvement). Renal findings responded completely to treatment in 31% of the patients, a partial response was observed in 4%, a stable course in 23.6% and progression in 38.5%. Amyloid storm was identified in nine patients and was found to be associated with increased mortality within 1 year.

**Conclusion:**

FMF patients still constitute the majority of AA amyloidosis patients in Turkey. The *MEFV* genotype and associated inflammatory load may affect the age of onset of AA amyloidosis, and earlier diagnosis and stricter follow-up and treatment may delay progression of the disease.

Rheumatology key messages

- FMF patients with two pathogenic *MEFV* variants develop AA amyloidosis 11 years earlier than those with one variant.
- A glomerular filtration rate <60 ml/min at admission predicts a higher end-stage renal disease risk in AA amyloidosis.
- Amyloid storm develops rarely (5.5%) but is associated with high mortality within 1 year.

## Introduction

Amyloid A (AA) amyloidosis is a protein-folding disorder associated with insoluble fibrillar aggregates of serum amyloid A (SAA) [1, 2]. Proteolytically cleaved and misfolded SAA monomers can form amyloid fibrils, which are deposited in extracellular spaces of various organs and result in functional damage [1, 2]. AA amyloidosis has been associated with increased SAA production due to uncontrolled activity of inflammatory disorders, including RA, AS, IBD and monogenic autoinflammatory disorders (AIDs) such as FMF, and TNF-receptor-associated periodic syndrome (TRAPS). FMF is the most common form of AID, mainly affecting people from the eastern Mediterranean, and has been a major cause of AA amyloidosis in Turkey [3–7]. The *MEFV* gene variations are responsible for FMF [8–10] and p.M694V, p.M680I and p.V726A in exon 10 are the most common pathogenic variants. Homozygosity for the p.M694V variant has been linked to a severe disease course and a higher AA amyloidosis risk [11–13] while patients with heterozygous pathogenic variants usually experience a relatively milder course [14].

The frequency of AA amyloidosis was reported to be as high as 20–30% in FMF patients in the past, but colchicine treatment resulted in a prominent decrease [15]. Several studies suggested the contribution of more penetrant *MEFV* variants, early disease onset, arthritis and erysipelas-like erythema attacks and the requirement for higher doses of colchicine to the tendency for AA amyloidosis [16–19].

In this study we aimed to evaluate clinical and laboratory characteristics, genotype–phenotype correlation, amyloid burden and outcome of patients with AA amyloidosis associated with FMF and other causes in our tertiary referral centre.

## Patients and methods

### Study group

Patients with an inflammatory rheumatic disease and biopsy-proven AA amyloidosis were included in this study. Patients without histopathologic confirmation and those with missing data were excluded. Biopsy sites were decided according to clinical and laboratory findings; however, subcutaneous adipose tissue, rectum, duodenum or gingiva was chosen for biopsy in some patients due to their easy access. The initial diagnosis of amyloidosis was made by Congo red stain and AA amyloidosis was confirmed by immunohistochemistry. Cardiac involvement was defined using non-invasive methods by demonstrating at least two of four echocardiography findings [decreased ejection fraction, increased cardiac septal wall thickness (CSWT) to >12 mm, increased granular echogenicity and/or amyloid valvulopathy and presence of left ventricular diastolic dysfunction], when a histopathologic confirmation of AA amyloidosis was available from other organs [20] and disorders such as cardiomyopathy, coronary artery disease and hypertension were excluded in the differential diagnosis. All FMF patients fulfilled the classification criteria described by Livneh *et al.* [21]. The amyloid storm was defined as proposed by Kukuy *et al.* [22] with items including an increase of creatinine and proteinuria values at least 2 times compared with the baseline and elevation of C-reactive protein (CRP) values >10 times compared with the highest normal level in <2 weeks.

Analyses of amyloid burden were conducted in only FMF-associated AA (FMF-AA) amyloidosis as the main and more homogeneous group of the study. Patients were divided into three groups according to their amyloid burden: G1 (only renal involvement), G2 [renal and gastrointestinal system (GIS) involvement] and G3 (renal, GIS and heart involvement).

Local ethics committee approval was obtained from the Istanbul Faculty of Medicine Ethics Committee (08.11.2019/19).

### Genetic evaluation

Available data for pathogenic exon 10 variants including p.M694V, p.M694I, p.M680I (G/C), p.M680I (G/A) and p.V726A were recorded from patients charts that included the results of targeted analyses of several of the most common mutations or sequencing data [23]. Exon 2 and 3 *MEFV* variants were not considered due to their lower penetrance and uncertain significance in regard to the FMF phenotype [23, 24].

### Treatment and outcomes

Management of all patients with AA amyloidosis was carried out in accordance with the current guidelines and tailored according to underlying diseases and renal functions. Colchicine was used at the highest tolerable dose (up to 2.5 mg/day) in FMF, and colchicine-refractory patients received one of the available anti-IL-1 treatments (anakinra or canakinumab). FMF patients experiencing an average of one or more attacks per month during the last 3 months or having continuously elevated acute phase reactants between attacks despite a maximum tolerable dose of colchicine were considered colchicine refractory. In accordance with local regulations, canakinumab was used in FMF patients who had an inadequate response to anakinra (up to 200 mg/day) or in cases who experienced intolerable adverse events. TNF inhibitors and anti-IL-17 secukinumab were used in patients with coexisting spondyloarthritis who had elevated acute phase reactants and/or persisting symptoms despite conventional treatments. Tocilizumab was used in patients with RA refractory to conventional DMARDs and in patients with FMF who had persisting chronic arthritis despite anti-TNF and/or anti-IL-1. The maximum colchicine dose was adjusted according to the estimated glomerular filtration rate (eGFR) in patients with renal failure, and patients continued to receive colchicine even if they started to use biologic DMARDs (b-DMARDs).

The eGFR was calculated by the Chronic Kidney Disease Epidemiology Collaboration formula [25]. Chronic renal failure (CRF) was defined as an eGFR <60 ml/min that persisted for at least 3 months. End-stage renal disease (ESRD) was defined as an eGFR ≤5 ml/min and/or requirement of renal replacement therapy such as dialysis or transplantation.

Responses to the treatment during the follow-up period were defined as follows: complete response: no increase in serum creatinine level with proteinuria <1 g/day; partial response: 50% decrease in the level of proteinuria with unchanged creatinine levels; stable disease: no significant change or worsening in serum creatinine and proteinuria after treatment; progressive disease: increase in serum creatinine and/or proteinuria levels, development of ESRD and/or development of additional new organ involvement. Amyloidosis outcome was categorized into two groups as favourable, which included complete and partial responses as well as a stable course, and poor, which included a progressive course and death.

### Statistical analyses

Descriptive statistics and discrete and continuous numerical variables were expressed as mean (s.d.) or median and interquartile range (IQR). Fisher’s exact and chi-squared tests were used to compare categorical variables with odds ratio (OR) calculations. Normally distributed parametric data were analysed with Student’s *t*-test and paired *t*-test and non-parametric data were analysed with Mann–Whitney U and Kruskal–Wallis tests. Multiple intergroup comparisons were made by Tukey post hoc analysis. Kaplan–Meier and logrank methods were used for survival analyses. Multivariate analysis was performed using logistic regression. Sensitivity and specificity calculations were performed using receiver operating characteristics (ROC) analysis. The Statistical Package for the Social Sciences (SPSS) version 21.0 (IBM, Armonk, NY, USA) was used for the analyses and a *P*-value <0.05 was considered significant.

## Results

### Baseline characteristics

Records of 184 AA amyloidosis patients followed up between 2000 and 2021 were identified and 174 were included in the analysis after the exclusion of 10 because of missing data. The characteristics of the patients are provided in Table 1. The most common underlying cause of AA amyloidosis was FMF (78.7%), followed by idiopathic cases (7.5%) and AS patients (4.6%) (Supplementary Table S1, available at *Rheumatology* online).

**Table 1.** Comparison of demographic and clinical and laboratory features of the study patients with AA amyloidosis

Characteristics	Total (*N* = 174)	FMF-AA amyloidosis (*n* = 137)	Non-FMF-AA amyloidosis (*n* = 37)	*P*-value [OR (95% CI)]
Age, years, median (IQR)	45 (20)	43 (20)	52 (20)	**0.014**
Male, *n* (%)	91 (52.6)	71 (51.8)	21 (56.8)	0.6
Disease duration, years, mean (s.d.)	34.4 (12.7)	36 (11.9)	23.5 (12.9)	**<0.001** ^a^
Age onset of FMF, years, median (IQR)	NA	7(4)	NA	
Age at diagnosis of amyloidosis, years, median (IQR)	31 (23)	29 (20)	45.5 (22)	**0.001**
Amyloidosis duration, years, median (IQR)	12.64 (11)	13 (11)	6.6 (12)	**0.003**
Family history of amyloidosis (*n* = 158), *n* (%)	42 (26.6)	38 (30.9)	5 (13.9)	**0.03 [4.1 (1.1, 7.7)]** ^b^
Family history of FMF (*n* = 160), *n* (%)	93 (58.1)	89 (71.2)	4 (11.1)	**<0.001 [41.4 (6.5, 60)]** ^b^
Creatinine, mg/dl, median (IQR)	0.8 (0.5)	0.8 (0.5)	0.9 (1)	0.3
eGFR, ml/min, median (IQR)	100.6 (62.3)	104.9 (63.2)	86.9 (71.9)	0.15
Proteinuria, g/day, median (IQR)	4.35 (5.65)	4 (6.2)	5 (5.3)	0.8
CRP, mg/l, median (IQR)	20 (20)	20 (18)	25 (35)	0.3
Organ involvement, *n* (%)				
Renal	170 (98.3)	134 (97.8)	37 (100)	0.36
Gastrointestinal	39 (22.5)	34 (24.8)	6 (16.2)	0.27
Heart	35 (20.2)	29 (21.2)	6 (16.2)	0.5
Liver	6 (3.5)	2 (1.5)	4 (10.8)	**0.006 [7.6 (1.4, 46.6)]** ^b^
Bone marrow	7 (4)	4 (2.9)	3 (8.1)	0.15
Thyroid	6 (3.5)	6 (4.4)	0	0.2
CRF at admission (*n* = 139), *n* (%)	75 (54)	61 (47.3)	14 (40)	0.4
ESRD at admission (*n* = 139), *n* (%)	25 (17.6)	22 (19.8)	3 (9.4)	0.17
ESRD development (overall), *n* (%)	83 (48.5)	71 (52.6)	13 (35.1)	**0.044 [3.5 (1.2, 4.4)]** ^b^
Renal transplantation, *n* (%)	59 (34.3)	53 (39)	7 (18.9)	**0.016 [5.2 (1.1, 6.7)]** ^b^
bDMARD use, *n* (%)	117 (68.4)	92 (67.2)	27 (73)	0.5
Anti-IL-1 therapy, *n* (%)	95 (81.2)	88 (62)	15 (40.5)	**0.009 [6.8 (1.2, 5.5)]**
Other bDMARD therapy, *n* (%)	21 (17.9)	6 (4.4)	15 (40.5)	**<0.001 [35.9 (5.2, 42.5)]** ^b^
Progressive course (*n* = 144), *n* (%)	42 (29.2)	30 (26.5)	12 (38.7)	0.19
Outcome, *n* (%)	102 (60.4)	83 (61.9)	19 (54.3)	0.4
Favourable	67 (39.6)	51 (38.1)	16 (45.7)	
Poor				
Mortality, *n* (%)	25 (14.5)	21 (15.3)	4 (10.8)	0.5

NA: not available.

Significant values are in bold.

a Independent *t*-test.

b Fischer’s exact test.

Baseline CRP, creatinine, eGFR and proteinuria levels did not differ between FMF-AA amyloidosis and non-FMF-AA amyloidosis patients. However, median age and age at the diagnosis of AA amyloidosis were significantly higher while the duration of underlying disease and duration of AA amyloidosis were significantly lower in patients with non-FMF-AA amyloidosis compared with FMF-AA amyloidosis patients (Table 1).

A total of 27% of the patients with AA amyloidosis had a family history of amyloidosis and 71% of the FMF-AA amyloidosis patients had a family history of FMF (Table 1). The diagnosis of AA amyloidosis was confirmed mainly by renal (75.5%) and GIS biopsies (10.9%) (Supplementary Table S2, available at *Rheumatology* online). Renal involvement was documented in 98.3% of the patients, while GIS involvement was documented in 22.5%, heart in 20.2%, bone marrow in 4% and thyroid and liver in 3.5% of patients (Supplementary Table S3, available at *Rheumatology* online).

Seventy-five patients (54%) had creatinine levels compatible with CRF and 34.3% had ESRD at the time of AA amyloidosis diagnosis. While liver involvement and the development of ESRD were significantly more frequent in patients with non-FMF-AA amyloidosis, involvement of other organs, frequency of CRF, ESRD at admission, requirement for bDMARD treatment and mortality rate did not differ between FMF-AA amyloidosis and non-FMF-AA amyloidosis (Table 1).

### Genotype–phenotype correlations

The distribution of the *MEFV* variants in patients with FMF and in non-FMF-AA amyloidosis patients is summarized in Supplementary Tables S4 and S5, respectively (available at *Rheumatology* online). The *MEFV* variant data were available in 87% (*n* = 119) of the patients with FMF. The most common pathogenic variant was p.M694V (91%) and the majority of these patients (74.1%) were homozygous. Overall, 69.7% of the patients with FMF had homozygous, 16.8% had compound heterozygous and 10% had heterozygous pathogenic variants (Supplementary Table S4, available at *Rheumatology* online).

The median patient age, age at diagnosis of FMF and age at diagnosis of AA amyloidosis were significantly lower and baseline eGFR levels were significantly higher in patients with two exon 10 *MEFV* variants compared with the patients with one variant (Table 2). Additionally, median age at the diagnosis of AA amyloidosis and baseline creatinine levels were significantly lower while the duration of amyloidosis and baseline eGFR levels were significantly higher in patients with a homozygous p.M694V variant compared with the patients with other *MEFV* variants (Table 2).

**Table 2.** *MEFV* gene variants and clinical correlations in patients with FMF-AA amyloidosis

Clinical and laboratory variables	Two exon 10 variants (*n* = 101)	One exon 10 variant (*n* = 12)	*P*-value [OR (95% CI)]	p.M694V homozygous (*n* = 80)	Other (*n* = 29)	*P*-value [OR (95% CI)]
Age, years, median (IQR)	40 (15)	52 (15)	**0.029**	39.5 (15)	47 (24)	0.095
Gender, *n* (%)						
Male (*n* = 62)	50 (49.5)	8 (66.7)	0.26	41 (51.2)	14 (48.3)	0.8
Female (*n* = 55)	51 (50.5)	4 (33.3)		39 (48.8)	15 (51.7)	
Age at onset of FMF, years, median (IQR)	7 (2)	7 (5)	0.18	7 (3)	7 (2)	0.5
Age at diagnosis of FMF, years, median (IQR)	23 (14)	35 (25)	**0.02**	23 (14)	25 (19)	0.36
Duration of FMF, years, mean (s.d.)	35 (11.5)	39.7 (8.4)	0.2^a^	34.3 (11)	41 (12)	0.054^a^
Age at diagnosis of amyloidosis, years, median (IQR)	27.5 (14)	41 (21)	**0.001**	27 (15)	35 (28)	**0.03**
Duration of amyloidosis, years, median (IQR)	13 (9.8)	10.8 (12.9)	0.2	13.9 (8.7)	8.7 (13)	**0.023**
Baseline CRP, mg/l, median (IQR)	20 (20)	25 (18)	0.4	20 (19)	15 (20)	0.9
Creatinine levels at diagnosis, mg/dl, median (IQR)	0.8 (0.5)	1.0 (2.8)	0.052	0.7 (0.6)	0.9 (0.5)	**0.035**
Proteinuria levels at diagnosis, g/day, median (IQR)	3.8 (5.9)	5 (14.8)	0.4	3 (4.75)	4.7 (12)	0.3
eGFR at diagnosis, ml/min, median (IQR)	106.4 (64.2)	79.1 (91.6)	**0.043**	110.9 (72.6)	83.1 (72.1)	**0.025**
Involved organs, *n* (%)						
Renal	99 (98)	12 (100)	0.6	79 (98.8)	28 (96.6)	0.45
Gastrointestinal	23 (22.8)	5 (41.7)	0.15	18 (22.5)	10 (34.5)	0.2
Heart	18 (17.5)	5 (41.7)	0.066	14 (17.5)	7 (24.1)	0.3
Liver	1 (1)	0	0.7	1 (1.3)	0	0.5
Bone marrow	3 (3)	0	0.5	3 (3.8)	0	0.3
Thyroid	5 (5)	0	0.6	5 (6.3)	0	0.2
CRF at admission (*n* = 109), *n* (%)	45 (45.9)	6 (54.5)	0.4	40 (51.9)	11 (39.3)	0.1
ESRD at admission (*n* = 93), *n* (%)	14 (17.3)	4 (33.3)	0.2	13 (21)	5 (18.5)	0.8
Development of ESRD (overall), *n* (%)	48 (48)	8 (66.7)	0.2	43 (54.4)	12 (41.4)	0.2
bDMARD requirement, *n* (%)	73 (72.3)	7 (58.3)	0.3	64 (80)	15 (51.7)	**0.003 [8.5 (1.5, 9.3)]**
Anti-IL-1 treatment, *n* (%)	70 (69.3)	7 (58.3)	0.4	61 (76.3)	15 (51.7)	**0.014 [6.1 (1.2, 7.3)]**
Progressive course (*n* = 26), *n* (%)	23 (25.6)	3 (33.3)	0.6	22 (30.6)	3 (12.5)	0.065^b^
Mortality, *n* (%)	10 (9.9)	3 (25)	0.1	7 (8.8)	5 (17.2)	0.2

Significant values in bold.

a Independent *t*-test.

b Fischer’s exact test.

**Figure 1.**
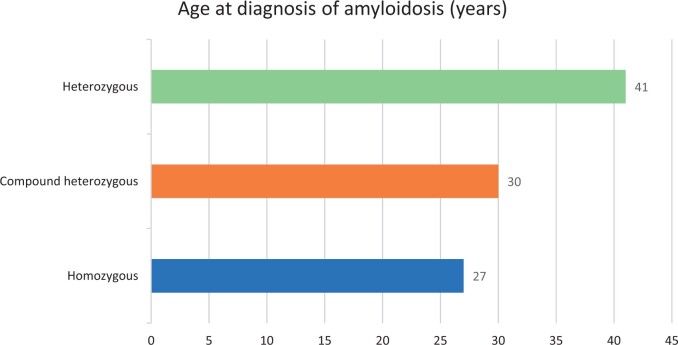
Comparison of age at the diagnosis of amyloidosis according to the *MEFV* gene exon 10 variant status in patients with FMF (*P* < 0.001 between the homozygous and heterozygous patients, *P* = 0.066 between the compound heterozygous and heterozygous patients and *P* = 0.3 between the homozygous and compound heterozygous patients)

The onset of AA amyloidosis was at least 11 years earlier in patients with homozygous or compound heterozygous variants compared with the heterozygous patients [median 27 years (IQR 14), 30 (27) and 41 (21), respectively] (Fig. 1). The frequency of cardiac involvement tended to be higher in patients with one pathogenic exon 10 variant compared with those with two variants. bDMARDs as well as anti-IL-1 were used significantly more frequently in patients with a homozygous p.M694V variant compared with the patients with other *MEFV* variants (Table 2).

A subset of the patients with non-FMF-AA amyloidosis (36.4%) had heterozygous *MEFV* variants without findings of FMF (Supplementary Table S5, available at *Rheumatology* online). Baseline creatinine was significantly higher in patients carrying heterozygous *MEFV* variants compared with those patients with none (Supplementary Table S6, available at *Rheumatology* online).

### Treatment response and outcome

Most FMF patients (94.5%) continued to take colchicine at a dose adjusted to eGFR values. A total of 119 (68.4%) patients used bDMARDs, and most were on IL-1 inhibitors (86.6%). Anakinra was used in 83.5%, canakinumab in 16.5% and other bDMARDs in 12.5%, and 4.2% used both anti-IL-1 and other bDMARDs at different time points (Supplementary Table S7, available at *Rheumatology* online). A decrease in the median CRP and proteinuria values was observed with bDMARDs, but creatinine values continued to increase along the course of the disease, and similar results were observed in both FMF-AA amyloidosis and non-FMF-AA amyloidosis (Supplementary Table S8, available at *Rheumatology* online).

During the follow-up, nearly half of the patients developed ESRD. Sixty patients (71.4%) with ESRD underwent renal transplantation and recurrence of renal amyloidosis was observed in 27.1% of them. Most of these patients with recurrent amyloidosis were referred to our clinic after the reappearance of proteinuria and none of them were on anti-IL-1 treatment before referral. The baseline creatinine values were significantly higher [1.2 mg/dl (s.d. 1.9) *vs* 0.8 (0.4)] and the proteinuria level tended to be higher [5.9 g (s.d. 5.6) *vs* 4 (5.65)] in the patients who developed ESRD compared with those who did not.

### Amyloid burden

The data from 137 FMF-AA amyloidosis patients were evaluated for amyloid burden (Supplementary Table S9, available at *Rheumatology* online) and 79 patients were classified as G1, 20 as G2 and 14 as G3. CSWT, troponin and pro-brain natriuretic peptide (BNP) levels were higher in G3 compared with G1 and G2, but troponin levels were not significantly different between G3 and G2. The overall mortality rate was 15.3% and the rate increased gradually in association with the amyloid burden (10% in G1, 15% in G2 and 43% in G3). There was a significant difference between the mortality rates of G3 and G1 (Supplementary Table S9 and Supplementary Fig. S1, available at *Rheumatology* online).

The number of exon 10 variants was lower in patients with a higher amyloid burden compared with those with a lower burden (Supplementary Table S9, available at *Rheumatology* online). Although the differences were more prominent for p.M694V homozygosity compared with others (72% and 93% in G1, 67% and 83% in G2 and 50% and 75% in G3, respectively), none reached statistically significant levels.

The number of involved organs was correlated with CSWT (*r* = 0.559), troponin (*r* = 0.646), pro-BNP (*r* = 0.572), baseline creatinine (*r* = 0.511) and baseline proteinuria levels (*r* = 0.321) and it was negatively correlated with baseline eGFR (*r* = −0.437) and the duration of bDMARD use (*r *= −0.235). The ROC analyses revealed 56% sensitivity and 70% specificity for the baseline creatinine [cut-off value (CoV) 0.95, area under the curve (AUC) 0.726]; 83.3% sensitivity and 74% specificity for troponin (CoV 35.5, AUC 0.864), 100% sensitivity and 85.5% specificity for pro-BNP (CoV 7246, AUC 0.897) and 79% sensitivity and 58% specificity for CSWT (CoV 11.5, AUC 0.727) in the prediction of the higher mortality rate (Supplementary Table S10, available at *Rheumatology* online).

### Treatment response

Overall, complete treatment response was observed in 31%, partial response in 4%, stable course in 23.6% and progression in 38.5% of the patients with AA amyloidosis (Supplementary Table S11, available at *Rheumatology* online). Response rates were not significantly different between FMF-AA amyloidosis and non-FMF-AA amyloidosis. A favourable outcome was seen in 61.9% of FMF-AA amyloidosis and 54.3% of non-FMF-AA amyloidosis patients (Supplementary Table S11, available at *Rheumatology* online). Overall, 25 patients (14.4%) died during the follow-up and the leading cause of death was infection (Supplementary Table S12, available at *Rheumatology* online). Most patients who died had more than one organ involved (56% and Supplementary Fig. S1, available at *Rheumatology* online). Although the mortality rate was higher in patients with FMF-AA amyloidosis (15.3%) compared with non-FMF-AA amyloidosis (10.8%), it did not reach statistical significance (Table 1).

### Factors associated with increased mortality

Univariate analysis showed that longer disease duration, older age at the diagnosis of AA amyloidosis, higher creatinine levels at baseline, frequency of CRF and ESRD at admission as well as development of ESRD at any time and cardiac involvement were associated with increased mortality. In the multivariate analysis, cardiac involvement and ESRD development at any time were found to be associated with increased mortality (Tables 3 and 4).

**Table 3.** Univariate and multivariate analyses of the factors associated with mortality in patients with AA amyloidosis

Clinical and laboratory variables	Univariate analysis			Multivariate analysis
	Died (*n* = 25)	Alive (*n* = 149)	*P*-value [OR (95% CI)	*P*-value [OR (95% CI)]
Age, years, median (IQR)	53.5 (31)	37 (18)	**0.003**	NS
Gender, *n* (%)				
Male (*n* = 92)	17 (68)	75 (50.3)	0.08	NS
Female (*n* = 82)	8 (32)	74 (49.7)		
Disease duration, years, mean (s.d.) (range)	42 (14.2) (25–56)	33.1 (11.8) (4–61)	**0.001** ^a^	NS
Age at diagnosis of amyloidosis, years, median (IQR)	37.5 (35)	29 (21)	**0.02**	NS
Amyloidosis duration, years, median (IQR)	9.8 (9.1)	9.8 (7.5)	0.6	
Creatinine, mg/dl, median (IQR)	1 (1.5)	0.75 (0.3)	**0.027**	NS
eGFR, ml/min, median (IQR)	71.3 (82.8)	107.1 (43.7)	**0.08**	NS
Proteinuria, g/day, median (IQR)	7 (7.75)	3.75 (4.9)	0.2	
CRP, mg/l, median (IQR)	14 (24)	19.5 (21)	0.7	
Organ involvement, *n* (%)				
Renal				
Yes (*n* = 171)	25 (100)	146 (98)	0.5	
No (*n* = 3)	0	3 (2)		
Gastrointestinal				
Yes (*n* = 40)	9 (36)	31 (20.8)	0.08	NS
No (*n* = 134)	16 (64)	118 (79.2)		
Heart				
Yes (*n* = 35)	10 (40)	25 (16.8)	**0.01 [7.2 (1.7, 10.2)]**	**0.037 [2.8 (1.07, 7.3)]**
No (*n* = 139)	15 (60)	124 (83.2)		
Liver				
Yes (*n* = 6)	2 (8)	4 (2.7)	0.2	
No (*n* = 168)	23 (92)	145 (97.3)		
Bone marrow				
Yes (*n* = 7)	3 (12)	4 (2.7)	0.06	NS
No (*n* = 167)	22 (88)	145 (97.3)		
Thyroid				
Yes (*n* = 6)	1 (4)	5 (3.4)	0.6	
No (*n* = 131)	24 (96)	144 (96.6)		
CRF at diagnosis, *n* (%)				
Yes (*n* = 75)	18 (72)	57 (41)	**0.004 [8.2 (1.45, 9.4)]** ^b^	NS
No (*n* = 89)	7 (28)	82 (59)		
ESRD at diagnosis, *n* (%)				
Yes (*n* = 25)	8 (42.1)	17 (13.7)	**0.006 [9.2 (1.6, 13)]** ^b^	NS
No (*n* = 118)	11 (57.9)	107 (86.3)		
ESRD development (total), *n* (%)				
Yes (*n* = 84)	20 (83.3)	64 (43.2)	**<0.001 [13.3 (2.1–20)]** ^b^	**0.002 (5.8; 1.86-18)**
No (*n* = 88)	4 (16.7)	84 (56.8)		
Renal transplantation, *n* (%)				
Yes (*n* = 60)	14 (56)	46 (31.1)	**0.016 [5.9 (1.2, 6.7)]** ^b^	NS
No (*n* = 24)	11 (44)	102 (68.9)		
Amyloidosis recurrence after renal transplantation, *n* (%)				
Yes (*n* = 16)	6 (46.2)	10 (21.7)	0.08	
No (*n* = 42)	7 (53.8)	35 (78.3)		
bDMARD treatment, *n* (%)				
Yes (*n* = 119)	17 (68)	102 (68.5)	0.6	
No (*n* = 55)	8 (32)	47 (31.5)		
Anti-IL-1 treatment, *n* (%)				
Yes (*n* = 103)	17 (68)	86 (57.7)	0.3	
No (*n* = 71)	8 (32)	63 (42.3)		

NS: non-significant.

Significant values in bold.

a Independent *t*-test.

b Fischer’s exact test.

**Table 4.** Mortality associated factors of patients with FMF-AA amyloidosis in multivariate analysis in a model giving 88.8%

Variable	β coefficient	s.e.	*P*-value [OR (95% CI)]
ESRD at diagnosis	2.18	1.05	**0.04 [8.9 (1.12, 71)]**
GIS involvement	1.72	0.8	**0.035 [5.6 (1.13, 27.7)]**
ESRD (overall)	2.83	0.9	**0.003 [17 (2.68, 107.6)]**
Heart involvement	2.55	0.8	**0.002 [12.8 (2.54, 64.9)]**
Diagnosis age of FMF	−0.04	0.02	0.08 [0.96 (0.91, 1.005)]
Constant	−1.69	1.24	0.175 (0.185)

Significant values in bold.

In FMF-AA amyloidosis patients, older age at the diagnosis of FMF, longer disease duration, older age at the diagnosis of AA amyloidosis, higher baseline creatinine level, lower eGFR at admission, GIS and cardiac involvement, CRF at the admission and ESRD development at any time were associated with higher mortality rates in univariate analysis (Supplementary Table S13, available at *Rheumatology* online). Similarly, higher GIS and cardiac involvement and ESRD development at any time were associated with increased mortality in FMF-AA amyloidosis patients in multivariate analysis (Table 3). A higher frequency of ESRD at admission in univariate analysis and renal transplantation in multivariate analysis were associated with increased mortality in non-FMF-AA amyloidosis (Supplementary Table S14, available at *Rheumatology* online).

**Figure 2.**
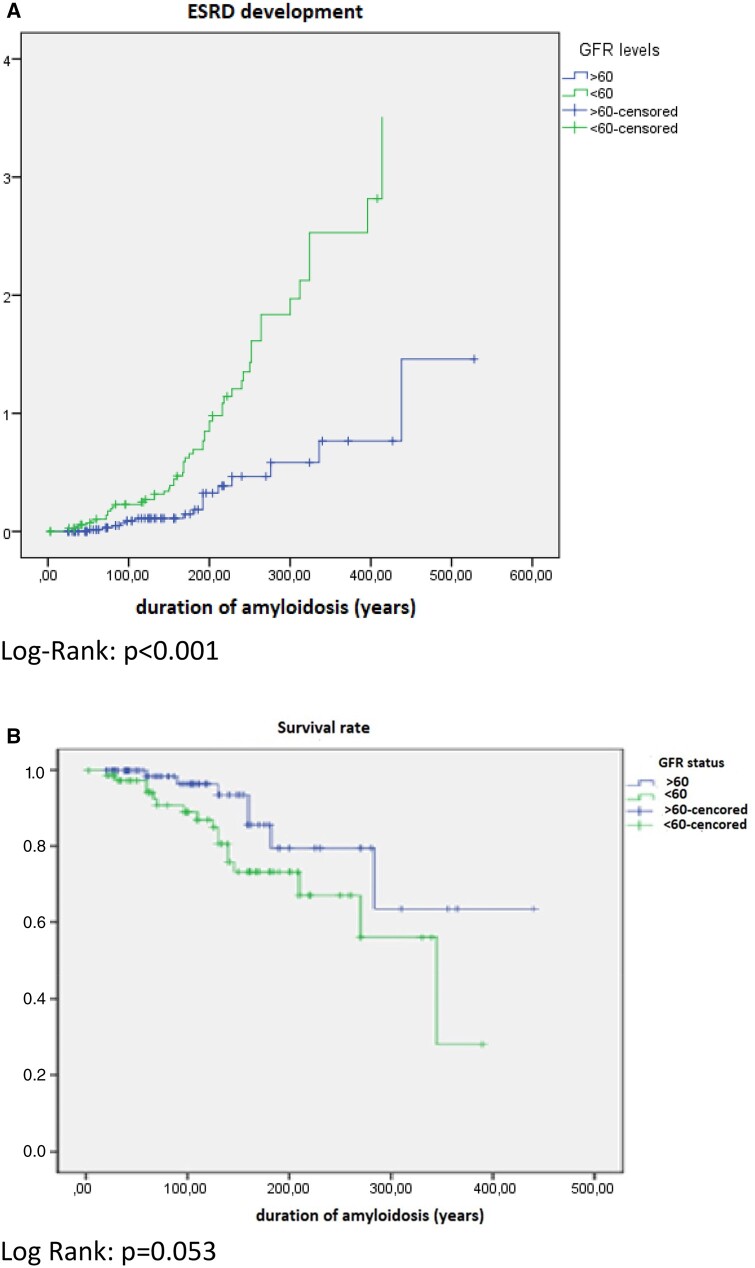
(A) Development of ESRD and **(B)** survival rate according to baseline eGFR in patients with AA amyloidosis

In Kaplan–Meier analysis, male gender and development of ESRD (Supplementary Figs S2 and S3, available at *Rheumatology* online) were associated with lower survival rates in patients with AA amyloidosis. Patients with a GFR <60 ml/min at admission had a higher frequency of progression to ESRD (Fig. 2A) and also showed a tendency towards higher mortality (Fig. 2B).

The mortality rate was higher in patients with one exon 10 *MEFV* variant compared with those with two variants (Supplementary Fig. S4, available at *Rheumatology* online), and there was a tendency for higher mortality in FMF patients with other *MEFV* variants compared with those with a homozygous p.M694V variant (Supplementary Fig. S5, available at *Rheumatology* online).

### Amyloid storm

The data from 164 patients allowed us to analyse the amyloid storm during follow-up, and 9 patients fulfilled the definition (Supplementary Table S15, available at *Rheumatology* online). Five patients had FMF-AA amyloidosis and four had non-FMF-AA amyloidosis (two AS, one idiopathic and one non-FMF AID). The median patient age and the age at diagnosis of FMF and amyloidosis were lower in patients who developed amyloid storm compared with those who did not, but the difference was not significant. All FMF patients with genotype data were homozygous for p.M694V and two non-FMF patients had one exon 10 variant (heterozygosity for p.M694V in one and a heterozygous variant of unknown significance, p.K695R, in another AS patient; none of the AS patients were on anti-IL-1 or colchicine). Amyloid storm was considered to be triggered by an infection in five patients, treatment non-adherence in three and surgery in one. Overall, 33% of AA amyloidosis patients with amyloid storm died, compared with 10% of the remaining patients. One of three patients died during the amyloid storm and the others died within 5 months (median) after the diagnosis of amyloid storm.

Univariate analysis revealed that involvement of three or more organs, bone marrow involvement and ongoing proteinuria were observed more frequently in patients with amyloid storm. ESRD development was also more frequent in patients with amyloid storm (67%) than in patients without (46%) (Supplementary Table S15, available at *Rheumatology* online). In multivariate analysis and survival analysis, amyloid storm was found to be associated with higher mortality (Supplementary Fig. S6, available at *Rheumatology* online).

## Discussion

AA amyloidosis is regarded as an important health problem despite the availability of more effective treatments for several inflammatory diseases [26]. This study retrospectively investigated a cohort of patients with AA amyloidosis from a single centre; among them, FMF was the most common underlying cause [3, 6, 27]. The distribution of underlying disorders does not reflect their prevalence in the population, which may suggest the involvement of certain inflammatory pathways in the pathogenesis of AA amyloidosis. Among others, AS has been listed as the second most commonly identified cause of AA amyloidosis, and an already established association between AS and FMF may support further importance of shared pathogenic pathways [28, 29]. A relatively high proportion of patients with no defined underlying inflammatory disorder emphasizes the need for additional studies to clarify pathogenic mechanisms leading to AA amyloidosis [30].

In this cohort we observed that median age and age at the diagnosis of AA amyloidosis were higher in patients with non-FMF-AA amyloidosis compared with FMF-AA amyloidosis patients, which may be due to the earlier onset of FMF. Although baseline levels of creatinine, GFR and frequency of CRF at admission did not differ between FMF-AA amyloidosis and non-FMF-AA amyloidosis patients, ESRD was observed more frequently in patients with FMF-AA amyloidosis. Lachmann *et al.* [1] reported a higher frequency of progression of AA amyloidosis and/or death in patients with monogenic AID and JIA compared with RA, which may be related with a higher production of SAA in AID compared with the rate in other disorders. Also, polymorphic SAA variants, such as the α/α genotype, have been associated with an increased risk for AA amyloidosis, and their contribution to the development and severity of the AA amyloidosis in FMF patients has been documented [31–33].

Kidneys are frequently affected in AA amyloidosis; however, the involvement of other organs, including the gastrointestinal tract and heart, have been documented in post-mortem investigations [34, 35]. The association of cardiac involvement has been considered an important feature of AL amyloidosis with poor prognosis [36] and has been reported rarely in AA amyloidosis. We herein documented the higher frequency of cardiac involvement in our cohort of AA amyloidosis patients with echocardiography as well as its association with higher mortality.

Documentation of non-renal AA amyloidosis in selected patients was a major limitation of our study. Therefore, a relatively lower frequency of bone marrow involvement in our cohort compared with the frequency of 79.5% reported by Sungur *et al.* [37] was possibly due to performing histopathologic investigations only in patients with serious haematological abnormalities. Similarly, histopathological investigation of the liver was conducted only in patients with relevant biochemical abnormalities. Lachmann *et al.* [1] used serum amyloid P (SAP) protein scintigraphy for the demonstration of hepatic involvement and reported that it was associated with ESRD but not increased mortality. We could not find any association between biopsy-proven hepatic involvement and ESRD or mortality, but we observed a higher frequency of liver involvement in non-FMF-AA amyloidosis compared with FMF-AA amyloidosis. The potential protective role of colchicine as well IL-1 inhibitors on liver function and/or prevention of fibrosis warrants further studies [38, 39].

In our cohort, p.M694V was the most common *MEFV* variant in patients with FMF-AA amyloidosis, consistent with previous studies [12, 40]. FMF patients with homozygous p.M694V or those with two exon 10 variants developed AA amyloidosis earlier than heterozygous patients, which emphasizes the importance of inflammatory activity associated with the number and penetrance of the *MEFV* variants [6, 12]. On the other hand, patients with two exon 10 variants had less amyloid burden and lower mortality rates compared with those with one variant, which may be due to closer follow-up and the use of more aggressive treatments with the expectation of worse prognosis. IL-1 inhibitors have been shown to be effective and safe in colchicine-refractory FMF patients [41–44]; there is no randomized controlled trial data in FMF-AA amyloidosis patients, but several case reports support their favourable effects [45–49].

Although the average CRP and proteinuria levels of the patients improved with treatment compared with the baseline values, deterioration of creatinine values and development of ESRD during follow-up showed that progression of amyloidosis continued in some patients, especially in those with CRF at admission. Ahbap *et al.* [27] and Lachmann *et al.*’s [1] similar findings of higher mortality and ESRD development rates in amyloidosis patients with higher baseline creatinine support our results and emphasize the importance of earlier diagnosis and starting effective treatments. These observations support that amyloid burden progresses with time, but this course may be accelerated in patients developing amyloid storm [22]. Data from the patients described in our cohort also confirmed the higher mortality rate associated with amyloid storm, and further studies are needed to clarify the underlying risk factors.

Monitoring SAA levels is the most sensitive follow-up method in AA amyloidosis, but this assay is not widely available. Using the more easily accessible CRP measurements for the assessment of acute phase response was an important limitation of this study. The retrospective design and a lack of proper control groups from non-amyloid patients and patients with other types of amyloidosis were also additional limitations.

In conclusion, in this cohort of AA amyloidosis patients from Turkey, the most common aetiology of AA amyloidosis was FMF. Progression of amyloidosis and an increased rate of ESRD development in patients with a GFR <60 ml/min at presentation emphasized the importance of early diagnosis of AA amyloidosis. Amyloid depositions progress with time and closer follow-up and stricter control of inflammatory findings with more effective treatments may help reduce morbidity and mortality.

## Supplementary Material

kead465_Supplementary_Data

## Data Availability

The dataset of the study is available from the corresponding author upon reasonable request.
