# Supplementary material for: Characteristics and course of patients with AA amyloidosis: single centre experience with 174 patients from Turkey
Source: Rheumatology (Oxford). 2023 Sep 20;63(2):319–28. doi: 10.1093/rheumatology/kead465 (PMC10836966; doi:10.1093/rheumatology/kead465)

**Supplementary Tables and Figures**

**Supplementary Table S1**. Underlying inflammatory disorders in the study patients with AA-a

| Underlying disorders | Number (%) |
| --- | --- |
| FMF | 137 (78.7) |
| Non-FMF | 37 (21.3) |
| *Idiopathic* | 13 (7.5) |
| *Ankylosing spondylitis* | 8 (4.6) |
| *Psoriatic arthritis* | 3 (1.7) |
| *Rheumatoid arthritis* | 2 (1.1) |
| *AOSD* | 2 (1.1) |
| *Takayasu* | 2 (1.1) |
| *DADA2* | 2 (1.1) |
| *TRAPS* | 1 (0.6) |
| *Gout* | 2 (1.1) |
| *GPA* | 1 (0.6) |
| *Crohn disease* | 1 (0.6) |
| *Behçet’s disease* | 1 (0.6) |

*AA-a: AA amyloidosis, FMF: Familial Mediterranean fever, TRAPS: TNF receptor associated periodic fever syndrome, DADA2: Deficiency of adenosine deaminase 2, GPA: Granulomatosis with polyangiitis, AOSD: Adult onset Still disease*

**Supplementary Table S2**: Sites of biopsies for the diagnosis of AA-a

|  | Number (n) | Frequency (%) |
| --- | --- | --- |
| Renal | 131 | 75.3 |
| Gastrointestinal system | 19 | 10.9 |
| Subcutaneous tissue | 12 | 6.8 |
| Thyroid | 3 | 1.7 |
| Gingiva | 3 | 1.7 |
| Bone marrow | 3 | 1.7 |
| Liver | 2 | 1.1 |
| Bladder | 1 | 0.6 |
| Total | 174 | 100 |

*AA-a: AA amyloidosis*

**Supplementary Table S3**: The organ involvement in patients with AA-a

|  | Number (n) | Frequency (%) |
| --- | --- | --- |
| Renal | 171 | 98.3 |
| Gastrointestinal system | 40 | 23 |
| Heart | 35 | 20.1 |
| Subcutaneous tissue | 16 | 9.2 |
| Bone marrow | 7 | 4 |
| Liver | 6 | 3.4 |
| Thyroid | 6 | 3.4 |
| Gingiva | 3 | 1.7 |
| Spleen | 1 | 0.6 |
| Lung | 1 | 0.6 |
| Urinary bladder | 1 | 0.6 |

*AA-a: AA amyloidosis*

**Supplementary Table S4**: The distribution of the *MEFV* gene exon 10 variants in patients with FMF

| MEFV Variants | Number (%) |
| --- | --- |
| M694V/M694V | 80 (67.2) |
| M694V/M680I | 11 (9.2) |
| M694V/V726A | 7 (6) |
| M680I/M680I | 2 (1.7) |
| M680I/V726A | 2 (1.7) |
| V726A/V726A | 1 (0.8) |
| M694V/- | 10 (8.4) |
| M680I/- | 1 (0.8) |
| V726A/- | 1 (0.8) |
| Negative* | 4 (3.4) |
| Not available | 18 (13) |

FMF: Familial Mediterranean Fever

* Those 4 patients with no exon 10 variant had typical FMF phenotype, but their limited targeted genetic analysis did not reveal any of the known variants at the time of analysis. Therefore, we could not exclude the presence of pathogenic variants in these patients.

**Supplementary Table S5**: The distribution of *MEFV* gene exon 10 variants in patients with non-FMF-AA-a

| MEFV Variants | Number (%) |
| --- | --- |
| Negative | 21 (56.8) |
| M694V/- | 8 (21.6) |
| V726A/- | 2 (5.4) |
| K695R/- | 2 (5.4) |
| Not available | 4 (10.8) |

Non-FMF-AA-a: AA-amyloidosis associated with disorders other than Familial Mediterranean Fever

**Supplementary Table S6**: The *MEFV* gene status and clinical correlation of patients with non-FMF-AA-a

| Clinical and laboratory variables | Carriage of MEFV variant (n=12) | Negative for MEFV variant (n=21) | P value | M694V  (n=8) | Others  (n=4) | P value |
| --- | --- | --- | --- | --- | --- | --- |
| Age (years), median (IQR) | 46 (17) | 53 (20) | 0.17 | 49 (17) | 42 (24) | 0.5 |
| Gender, n (%)  Male  Female | 10 (50)  2 (15.4) | 10 (50)  11 (84.6) | 0.055 | 7 (87.5)  1 (12.5) | 3 (75)  1 (25) | 1 |
| Duration of underlying disease (years), mean±SD (range) | 21.4±9 (9-37) | 22.7±10 (7-36) | 0.9 | 24.5 (17) | 16 (14) | 0.2 |
| Age at diagnosis of amyloidosis (years), median (IQR) | 36.5 (18) | 47 (28) | 0.4 | 41 (20) | 34 (19) | 0.3 |
| Duration of amyloidosis (years), median (IQR) | 6.5 (12) | 8 (12.3) | 0.6 | 5.6 (14.4) | 6.5 (7) | 0.8 |
| Baseline CRP (mg/L),  median (IQR) | 27.5 (57) | 19.5 (31) | 0.6 | 16.95 (51) | 52.5 (29) | 0.4 |
| Creatinine levels at diagnosis (mg/dL), median (IQR) | 1 (2.1) | 0.8 (0.4) | ***0.049**** | 1.65 (2.1) | 1 (3.5) | 1 |
| Proteinuria levels at diagnosis (g/day), median (IQR) | 6 (4.6) | 3 (4.7) | 0.1 | 5.9 (5.6) | 6.2 (27) | 0.6 |
| e-GFR at diagnosis (ml/min),  median (IQR) | 68.6 (82.3) | 100.6 (48.2) | 0.16 | 49.4 (68.8) | 88.2 (72) | 0.7 |
| Organ involvement, n (%)  Renal  GIS  Heart  Liver  Bone marrow  Thyroid | 12 (100)  2/17 (16.2)  1/4 (25)  0  1/12 (8.3)  No case | 21 (100)  2 (9.5)  2/10 (20)  3 (100)  0  No case | NA  0.6  1  0.2  0.4  - | 8 (100)  2 (25)  1/3 (33.3)  No case  0  No case | 4 (100)  0  0  No case  1 (25)  No case | NA  0.5  1  -  0.3  - |
| CRF at diagnosis, n (%) | 5 (41.7) | 6 (31.6) | 0.5 | 4 (50) | 1 (25) | 0.6 |
| ESRD at diagnosis, n (%) | 0 | 3/19 (15.8) | 0.5 | 0 | 0 | NA |
| Development of ESRD (overall), n (%) | 7 (58.3) | 6 (28.6) | 0.09 | 5 (62.5) | 2 (50) | 1 |
| Renal transplantation, n (%) | 4 (33.3) | 3 (14.3) | 0.2 | 3 (37.5) | 1 (25) | 1 |
| b-DMARD requirement, n (%) | 10 (83.3) | 14 (66.7) | 0.3 | 6 (75) | 4 (100) | 0.5 |
| Anti-IL-1 treatment, n (%) | 6 (50) | 9 (43) | 0.7 | 3 (37.5) | 3 (75) | 0.5 |
| Progressive course, n (%) | 5/10 (50) | 7/18 (39) | 0.7 | 4 (50) | 1/2 (50) | 1 |
| Mortality, n (%) | 2 (16.7) | 2 (9.5) | 0.6 | 0 | 2 (50) | 0.09 |

*FMF-AA-a: Familial Mediterranean Fever-associated AA-amyloidosis, IQR: Interquartile range, SD: Standard deviation, CI: Confidence interval, e-GFR: Estimated glomerular filtration rate, CRP: C-reactive protein, GIS: Gastrointestinal system, CRF: Chronic renal failure, ESRD: End stage renal disease, NA: Not available*

*Mann Whitney U test

**^Ɨ^** Fischer’s exact test

**Supplementary Table S7:** Biological treatment of patients with AA-a

| Biological treatment | Numbers (n) | Percent (%) |
| --- | --- | --- |
| Anakinra | 86 | 72.3 |
| Canakinumab | 17 | 14.3 |
| Etanercept | 7 | 4 |
| Tocilizumab | 5 | 2.9 |
| Infliximab | 4 | 2.3 |
| Secukinumab | 3 | 1.7 |
| Adalimumab | 2 | 1.1 |
| Total | 119 | 100 |

**Supplementary Table S8:** Laboratory changes of patients with AA-a during the follow-up

| Variable | Total (n=174) | FMF-AA-a  (n=137) | Non-FMF-AA-a  (n=37) |
| --- | --- | --- | --- |
| Creatinine (mg/dL), median (IQR)  Baseline  After treatment  p value* | 0.8 (0.5)  1.1 (1.2)  ***<0.001*** | 0.8 (0.5)  1 (1.1)  ***<0.001*** | 0.9 (1)  1.4 (0.8)  0.25 |
| Proteinuria (g/day), median (IQR)  Baseline  After treatment  p value* | 4.35 (5.65)  0.85 (3.1)  ***<0.001*** | 4 (6.2)  0.5 (2.8)  ***<0.001*** | 5 (5.3)  1.1 (1.2)  ***0.005*** |
| CRP (mg/L), median (IQR)  Baseline  After treatment  p value* | 20 (20)  2.8 (7.1)  ***<0.001*** | 20 (18)  2.7 (6.6)  ***<0.001*** | 25 (35)  3 (12.3)  ***0.001*** |

*AA-a: AA amyloidosis, FMF: Familial Mediterranean fever, IQR: Interquartile range, CRP: C-reactive protein*

* Mann Whitney U test

**Supplementary Table S9**. Univariate and correlation analyses of clinical and laboratory features according to the amyloid burden

| Variables | Amyloid burden | | | | | | Correlation analysis | | |  |
| --- | --- | --- | --- | --- | --- | --- | --- | --- | --- | --- |
|  | **1 organ** | **≥2 organs** | **P value (OR;**  **95% CI)** | **<3 organ** | **≥3 organ** | **P value (OR;**  **95% CI)** | | **r** | **p value** | |
| Age (years), mean±SD | 43.1±12.6 | 47.1±11.7 | 0.06 | 44.1±12.7 | 47.8±10.6 | 0.2 | |  |  | |
| Gender, male, n (%) | 37 (45.7) | 38 (68) | ***0.01***  ***(6.6; 1.2-5.1)*** | 63 (55.3) | 12 (52.2) | 0.8 | |  |  | |
| Age of FMF onset (years), median (IQR) | 7 (2) | 7 (5) | 0.55* | 7 (3) | 7 (7) | 0.5* | | 0.103 | 0.2 | |
| Duration of FMF (years), mean±SD | 32.9±11.8 | 36.3±12.2 | 0.1 | 34.1±12.3 | 35.1±10.9 | 0.8 | |  |  | |
| Age at diagnosis of amyloidosis (years), mean±SD | 31.1±13.9 | 34.1±13 | 0.2 | 31.9±13.5 | 34.6±14 | 0.4 | | 0.165 | ***0.034*** | |
| Duration of amyloidosis (months), mean±SD | 162±103 | 161±91 | 0.96 | 161±100 | 162±84 | 1 | |  |  | |
| Baseline CRP (mg/L), mean±SD | 20.5±12.5 | 18.6±15 | 0.5 | 20.1±13.8 | 18±13 | 0.5 | | 0.071 | 0.4 | |
| Baseline proteinuria (g/day), median (IQR) | 3.6 (5.6) | 5 (9.3) | ***0.04**** | 4 (6) | 6.5 (7.3) | 0.1 | | 0.321 | ***0.008*** | |
| Baseline creatinine (mg/dL), mean±SD | 0.8±0.4 | 1.6±1 | ***<0.001*** | 0.95±0.6 | 2.2±1.5 | ***<0.001*** | | 0.511 | ***<0.001*** | |
| Baseline e-GFR (ml/min), mean±SD | 104.8±30.7 | 70.3±42.7 | ***<0.001*** | 97.5±36 | 52±39 | ***0.004*** | | -0.437 | ***<0.001*** | |
| Chronic renal failure at admission, n (%) | 25 (32.5) | 36 (69.2) | ***<0.001***  ***(16.8; 2.2-10)*** | 43 (40) | 18 (86) | ***<0.001***  ***(14.9; 2.5-32.6)*** | |  |  | |
| ESRD at admission, n (%) | 7 (10.8) | 15 (32.6) | ***0.004***  ***(8.1; 1.5-10.9)*** | 12 (13) | 10 (55.6) | ***<0.001***  ***(17.3; 2.8-25.6)*** | |  |  | |
| Ejection fraction, mean±SD | 65±7.2 | 63.3±12.8 | 0.4 | 64.7±8 | 62.2±14.6 | 0.3 | | -0.074 | 0.5 | |
| Left ventricular wall thickness (cm), mean±SD | 4.3±0.5 | 4.5±0.7 | ***0.03*** | 4.4±0.5 | 4.43±0.97 | 0.8 | | 0.133 | 0.2 | |
| Cardiac septal wall thickness (mm), mean±SD | 10.1±1.6 | 12.8±2.3 | ***<0.001*** | 11±2.2 | 13.2±2.1 | ***<0.001*** | | 0.559 | ***<0.001*** | |
| Troponin (pg/mL), median (IQR) | 7.5 (11.6) | 72 (102) | ***<0.001**** | 10 (21.7) | 73.5 (89) | ***0.002**** | | 0.646 | ***<0.001*** | |
| pro-BNP (pg/mL), median (IQR) | 168 (914) | 1590 (14033) | ***0.002**** | 316 (1186) | 4968 (14143) | ***0.002**** | | 0.572 | ***<0.001*** | |
| MEFV variant (n, %) |  |  |  |  |  |  | |  |  | |
| One copy | 5 (35.7) | 9 (64.3) | 0.09 | 9 (64.3) | 5 (35.7) | ***0.044 (3.5; 1.03-12)*** | |  |  | |
| Two copies | 63 (61.2) | 40 (38.8) |  | 89 (86.4) | 14 (13.6) |  | |  |  | |
| MEFV variant (n, %) |  |  |  |  |  |  | |  |  | |
| M694V homozygous | 48 (60) | 32 (40) | 0.5 | 69 (86.3) | 11 (13.8) | 0.3 | |  |  | |
| Other MEFV variant | 20 (54) | 17 (46) |  | 29 (78.4) | 8 (21.6) |  | |  |  | |
| ESRD development (overall), n (%) | 33 (41.8) | 38 (68) | ***0.003***  ***(8.9; 1.4-6)*** | 50 (44.6) | 21 (91.3) | ***<0.001***  ***(16.7; 2.9-58)*** | |  |  | |
| Duration of b-DMARD (months), mean±SD | 70.7±36.7 | 60.7±34.8 | 0.2 | 68.4±36 | 55.4±34 | 0.18 | | -0.235 | ***0.03*** | |
| Mortality, n (%) | 8 (9.9) | 13 (23.2) | ***0.038***  ***(4.5; 1.06-7.2)*** | 13 (11.4) | 8 (34.8) | ***0.005***  ***(8; 1.5-11.7)*** | |  |  | |

*IQR: Interquartile range, SD: Standard deviation, FMF: Familial Mediterranean Fever, CI: Confidence interval, e-GFR: Estimated glomerular filtration rate, CRP: C-reactive protein, CRF: Chronic renal failure, ESRD: End stage renal disease, b-DMARD: Biological disease modifying anti-rheumatic drug*

*Mann Whitney U test

**^Ɨ^** Fischer’s exact test

**Supplementary Table S10:** Performance of clinical and laboratory features of patients in amyloidosis burden and mortality (ROC analysis)

| Variable | ≥2 organ involvement | | | | ≥3 organ involvement | | | | Mortality | | | |  |
| --- | --- | --- | --- | --- | --- | --- | --- | --- | --- | --- | --- | --- | --- |
|  | **AUC (LR)** | **Cut-off** | **Sensitivity /specificity** | **P value**  **(95% CI)** | **AUC (LR)** | **Cut-off** | **Sensitivity /specificity** | **P value**  **(95% CI)** | **AUC (LR)** | **Cut-off** | **Sensitivity /specificity** | **P value**  **(95 % CI)** | |
| Creatinine | 0.785 (3.25) | 0.95 | 62.5/81 | ***<0.001 (0.67-0.9)*** | 0.808 (2.2) | 0.95 | 66.7/70 | ***0.013***  ***(0.64-0.98)*** | 0.726 (1.9) | 0.95 | 56/70 | ***0.03***  ***(0.56-0.9)*** | |
| Troponin | 0.871 (9.1) | 19.5 | 76.2/91.7 | ***<0.001 (0.75-0.99)*** | 0.817 (3.85) | 24 | 92/76.2 | ***0.003***  ***(0.67-0.97)*** | 0.864 (3.2) | 35.5 | 83.3/74.1 | ***0.006***  ***(0.73-0.99)*** | |
| pro-BNP | 0.791 (3.2) | 1008 | 63.6/80 | ***0.003***  ***(0.65-0.94)*** | 0.804 (3.1) | 1116 | 77/75 | ***0.003***  ***(0.67-0.94)*** | 0.897 (6.3) | 7246 | 100/85.5 | ***0.024***  ***(0.79-1.0)*** | |
| CSWT | 0.817 (6.5) | 12.5 | 55/91.5 | ***<0.001 (0.74-0.9)*** | 0.780 (3.3) | 12.5 | 69.6/78.7 | ***<0.001 (0.68-0.88)*** | 0.727 (1.9) | 11.5 | 78.6/58.3 | ***0.007***  ***(0.61-0.84)*** | |
| Amyloid burden |  |  |  |  |  |  |  |  | 0.664 (1.7) | 1.5 | 62/63 | ***0.017***  ***(0.53-0.8)*** | |

AUC: Area under curve, CI: Confidence interval, LR: Likelihood ratio, CSWT: Cardiac septal wall thickness, LR: Likelihood ratio

**Supplementary Table S11:** Treatment responses and outcomes of patients with AA-a

| Amyloidosis response, n (%) | Total (n=174) | FMF-AA-a (n=137) | Non-FMF-AA-a (n=37) | P value |
| --- | --- | --- | --- | --- |
| Complete response | 54 (31) | 47 (34.3) | 7 (18.9) | 0.07 |
| Partial response | 7 (4) | 6 (4.4) | 1 (2.7) | 0.5 |
| Stable course | 41 (23.6) | 30 (21.9) | 11 (29.7) | 0.3 |
| Progressive course | 67 (38.5) | 51 (37.2) | 16 (43.2) | 0.5 |
| Lost to the follow up | 5 (2.9) | 3 (2.2) | 2 (5.4) | 0.7 |
| Amyloidosis outcome, n (%) |  |  |  |  |
| Favorable outcome | 102 (60.4) | 83 (61.9) | 19 (54.3) | 0.4 |
| Poor outcome | 67 (39.6) | 51 (38.1) | 16 (45.7) |  |

*AA-a: AA amyloidosis, FMF: Familial Mediterranean fever*

**Supplementary Table S12**: Reported causes of mortality in patients with AA-a

| Causes | Number (n) | % |
| --- | --- | --- |
| Sepsis | 6 | 24 |
| Pneumonia | 3 | 12 |
| GIS perforation | 1 | 4 |
| Heart failure | 1 | 4 |
| Brain tumor | 1 | 4 |
| Myocardial infarction | 1 | 4 |
| Unknown | 12 | 48 |
| Total | 25 | 100 |

AA-a: AA amyloidosis, GIS: Gastrointestinal system

**Supplementary Table S13**: Univariate and multivariate analyses of the associated factors of mortality in patients with FMF- AA-a

| Clinical and laboratory variables | Univariable analysis | | | Multivariable analysis |
| --- | --- | --- | --- | --- |
|  | **Died** | **Alive** | **P value**  **(OR; 95% CI)** | **P value**  **(OR; 95% CI)** |
| Age (years), median (IQR) | 53.5 (31) | 37 (20) | ***0.001*** | NS |
| Gender |  |  |  |  |
| Male (n=71) | 13 (61.9) | 58 (50) | 0.3 |  |
| Female (n=66) | 8 (38.1) | 58 (50) |  |  |
| Disease duration (years), mean±SD (range) | 42 (25-56) | 33.1 (4-61) | ***<0.001**** |  |
| Age onset of FMF (years), median (IQR) | 7 (3) | 7 (4) | 0.15 |  |
| Age at diagnosis of FMF (years), median (IQR) | 34 (34) | 24.5 (20) | ***0.005*** | 0.08  (0.91-1.005) |
| Age at diagnosis of amyloidosis (years), median (IQR) | 37.5 (35) | 29 (21) | ***0.009*** |  |
| Amyloidosis duration (years), median (IQR) | 9.8 (9.1) | 9.5 (7.6) | 0.65 |  |
| Creatinine (mg/dL), median (IQR) | 1 (1.5) | 0.75 (0.3) | ***0.03*** |  |
| e-GFR (ml/min), median (IQR) | 71.3 (82.8) | 106.6 (43) | ***0.035*** |  |
| Proteinuria (g/day), median (IQR) | 7 (7.75) | 3.75 (5.2) | 0.3 |  |
| CRP (mg/L), median (IQR) | 14 (24) | 20 (20) | 0.5 |  |
| Organ involvement, n (%) |  |  |  |  |
| Renal |  |  |  |  |
| Yes (n=134) | 21 (100) | 113 (97.4) | 0.5 |  |
| No (n=3) | 0 | 3 (2.6) |  |  |
| Gastrointestinal |  |  |  |  |
| Yes (n=34) | 9 (42.9) | 25 (21.6) | ***0.04***  ***(4.3; 2.7-7.2)* ^Ɨ^** | ***0.035***  ***(5.6; 1.13-27.7)*** |
| No (n=103) | 12 (57.1) | 91 (78.7) |  |  |
| Heart |  |  |  |  |
| Yes (n=29) | 10 (47.6) | 19 (16.4) | ***0.003***  ***(10.4; 1.5-11.7)* ^Ɨ^** | ***0.002***  ***(12.8; 2.54-64.9)*** |
| No (n=108) | 11 (52.4) | 97 (83.6) |  |  |
| Liver |  |  |  |  |
| Yes (n=2) | 1 (4.8) | 1 (0.9) | 0.28 |  |
| No (n=135) | 20 (95.2) | 115 (99.1) |  |  |
| Bone marrow |  |  |  |  |
| Yes (n=4) | 2 (9.5) | 2 (1.7) | 0.1 |  |
| No (n=133) | 19 (90.5) | 114 (98.3) |  |  |
| Thyroid |  |  |  |  |
| Yes (n=6) | 1 (4.8) | 5 (4.3) | 0.6 |  |
| No (n=131) | 20 (95.2) | 111 (95.7) |  |  |
| CRF at diagnosis, n (%) |  |  |  |  |
| Yes (n=61) | 15 (71.4) | 46 (42.6) | ***0.014***  ***(5.9; 1.2-9.4)* ^Ɨ^** | NS |
| No (n=68) | 6 (28.6) | 62 (57.4) |  |  |
| ESRD at diagnosis, n (%) |  |  |  |  |
| Yes (n=22) | 6 (35.3) | 16 (17) | 0.08 | ***0.04***  ***(8.9; 1.12-197)*** |
| No (n=89) | 11 (64.7) | 78 (83) |  |  |
| ESRD development (overall), n (%) |  |  |  |  |
| Yes (n=71) | 17 (85) | 54 (47) | ***0.001***  ***(9.9; 1.8-23)* ^Ɨ^** | ***0.003***  ***(16.98; 2.68-107.6)*** |
| No (n=64) | 3 (15) | 61 (53) |  |  |
| Renal transplantation, n (%) |  |  |  |  |
| Yes (n=53) | 11 (52.4) | 42 (36.5) | 0.2 |  |
| No (n=18) | 6 (47.6) | 73 (63.5) |  |  |
| Amyloidosis recurrence after Renal transplantation, n (%) |  |  |  |  |
| Yes (n=15) | 5 (45.5) | 10 (23.8) | 0.25 |  |
| No (n=37) | 6 (54.5) | 32 (76.2) |  |  |
| MEFV gene status, n (%) |  |  |  |  |
| One variant (n=12) | 3 (23.1) | 9 (9) | 0.1 |  |
| Two variants (n=101) | 10 (76.9) | 91 (91) |  |  |
| MEFV gene status, n (%) |  |  |  |  |
| M694V homozygous (n=80) | 7 (58.3) | 73 (75.3) | 0.2 |  |
| Other variants (n=29) | 5 (41.7) | 24 (24.7) |  |  |
| b-DMARD treatment, n (%) |  |  |  |  |
| Yes (n=92) | 14 (66.7) | 78 (67.2) | 0.9 |  |
| No (n=45) | 7 (33.3) | 38 (32.8) |  |  |
| Anti-IL-1 treatment, n (%) |  |  |  |  |
| Yes (n=88) | 14 (66.7) | 74 (63.8) | 0.8 |  |
| No (n=49) | 7 (33.3) | 42 (36.2) |  |  |

*AA-a: AA amyloidosis, FMF: Familial Mediterranean fever, IQR: Interquartile range, SD: Standard deviation, CI: Confidence interval, e-GFR: Estimated glomerular filtration rate, CRP: C-reactive protein, GIS: Gastrointestinal system, CRF: Chronic renal failure, ESRD: End stage renal disease, NS: Non-significant*

* Independent t test, **^Ɨ^** Fischer’s exact test

**Supplementary Table S14:** Univariate and multivariate analysis of associated factors of mortality in patients with non-FMF AA-a

| Clinical and laboratory variables | Univariate analysis | | | Multivariable analysis | |
| --- | --- | --- | --- | --- | --- |
|  | **Died** | **Alive** | **P value**  **(OR; 95% CI)** | **P value**  **(OR; 95% CI)** | |
| Age (years), median (IQR) | 56 (16) | 51.5 (21) | 0.6 |  |  |
| Gender, n (%) |  |  |  |  |  |
| Male (n=21) | 4 (100) | 17 (51.5) | 0.1 | NS | |
| Female (n=16) | 0 | 16 (48.5) |  |  |  |
| Disease duration (years), mean±SD (range) | 19±7.1 (14-24) | 26±13.4 (19.5) | 0.5 |  |  |
| Age at diagnosis of amyloidosis (years), median (IQR) | 40.5 (21) | 45 (28) | 0.98 |  |  |
| Amyloidosis duration (years), median (IQR) | 9.8 (10.8) | 7 (13) | 0.9 |  |  |
| Creatinine (mg/dL), median (IQR) | NA | 0.84 (1.1) |  |  |  |
| e-GFR (ml/min), median (IQR) | NA | 83.9 (74.4) |  |  |  |
| Proteinuria (g/day), median (IQR) | 6.15 | 4.5 (5.4) | 0.5 |  |  |
| CRP (mg/L), median (IQR) | NA | 26 (38) |  |  |  |
| Organ involvement, n (%) |  |  |  |  |  |
| Renal |  |  |  |  |  |
| Yes (n=37) | 4 (100) | 33 (100) | NA |  |  |
| No (n=0) |  |  |  |  |  |
| GIS |  |  |  |  |  |
| Yes (n=6) | 0 | 6 (18.2) | 1 |  |  |
| No (n=31) | 4 (100) | 27 (81.8) |  |  |  |
| Heart |  |  |  |  |  |
| Yes (n=6) | 0 | 6 (18.2) | 1 |  |  |
| No (n=31) | 4 (100) | 27 (81.8) |  |  |  |
| Liver |  |  |  |  |  |
| Yes (n=4) | 1 (25) | 3 (9.1) | 0.3 |  |  |
| No (n=33) | 3 (75) | 30 (90.9) |  |  |  |
| Bone marrow |  |  |  |  |  |
| Yes (n=3) | 1 (25) | 2 (6.1) | 0.3 |  |  |
| No (n=34) | 3 (75) | 31 (93.9) |  |  |  |
| Thyroid |  |  |  |  |  |
| Yes (n=0) |  |  | NA |  |  |
| No (n=37) | 4 (100) | 33 (100) |  |  |  |
| CRF at admission, n (%) |  |  |  |  |  |
| Yes (n=14) | 3 (75) | 11 (35.5) | 0.3 |  |  |
| No (n=21) | 1 (25) | 20 (64.5) |  |  |  |
| ESRD at admission, n (%) |  |  |  |  |  |
| Yes (n=3) | 2 (100) | 1 (3.3) | ***0.006***  ***(20.6; 2.3-8.9)* ^Ɨ^** | NS | |
| No (n=29) | 0 | 29 (96.7) |  |  |  |
| ESRD development (overall), n (%) |  |  |  |  |  |
| Yes (n=13) | 3 (75) | 10 (30.3) | 0.077 | NS | |
| No (n=24) | 1 (25) | 23 (69.7) |  |  |  |
| Renal transplantation, n (%) |  |  |  |  |  |
| Yes (n=7) | 3 (75) | 4 (12.1) | **0.016**  **(9.2; 1.8-263) ^Ɨ^** | ***0.015***  ***(21.8; 1.8-263*** | |
| No (n=6) | 1 (25) | 29 (87.9) |  |  |  |
| Amyloidosis recurrence after Renal transplantation, n (%) |  |  |  |  |  |
| Yes (n=1) | 1 (50) | 0 | 0.3 |  |  |
| No (n=5) | 1 (50) | 4 (100) |  |  |  |
| b-DMARD treatment, n (%) |  |  |  |  |  |
| Yes (n=27) | 3 (75) | 24 (72.7) | 1 |  |  |
| No (n=10) | 1 (25) | 9 (27.3) |  |  |  |

*AA-a: AA amyloidosis, FMF: Familial Mediterranean fever, IQR: Interquartile range, SD: Standard deviation, CI: Confidence interval, e-GFR: Estimated glomerular filtration rate, CRP: C-reactive protein, GIS: Gastrointestinal system, CRF: Chronic renal failure, ESRD: End stage renal disease, NS: Non-significant, NA: Not available*

**^Ɨ^** Fischer’s exact test

**Supplementary Table S15**: Comparison of clinical and laboratory features of AA-a patients with and without amyloid storm.

| Variables | Patients with amyloid storm (n=9) | Other patients  (n=155) | P value  (OR; 95% CI) |
| --- | --- | --- | --- |
| Age (years), median (IQR) | 37 (10) | 45 (20) | 0.2 |
| Gender, male, n (%) | 4 (44) | 86 (56) | 0.7 |
| Diagnosis, n (%) |  |  |  |
| FMF | 5 (4) | 122 (96) | 0.1 |
| Non-FMF | 4 (11) | 33 (89) |  |
| Organ distribution, n (%) |  |  |  |
| Renal | 9 (100) | 152 (98) | 1 |
| Gastrointestinal | 4 (44) | 33 (21) | 0.1 |
| Heart | 2/6 (33) | 32/111 (29) | 1 |
| Liver | 1 (11) | 5 (3) | 0.3 |
| Bone marrow | 2 (22) | 5 (3) | ***0.049***  ***(7.4; 1.4-52) ^Ɨ^*** |
| Diagnosis age of FMF (years), median (IQR) | 17.5 (30) | 24 (20) | 0.3 |
| Diagnosis age of amyloidosis (years), median (IQR) | 26 (26) | 31 (22) | 0.2 |
| Duration of amyloidosis (years), median (IQR) | 10 (14.6) | 11.2 (11) | 1 |
| Number of organ involvement, median (IQR) | 2 (2) | 1 (1) | 0.09 |
| ≥3 organ involvement, n (%) | 3/5 (60) | 20/122 (16) | ***0.04***  ***(4.3; 0.92-68)* ^Ɨ^** |
| Baseline laboratory values, median (IQR) |  |  |  |
| CRP (mg/L) | 10 (8) | 20 (20) | 0.06 |
| Proteinuria (g/day) | 5.3 (4.3) | 4 (5.7) | 0.9 |
| Creatinine (mg/dL) | 0.8 (0.6) | 0.8 (0.6) | 0.6 |
| e-GFR (ml/min) | 95 (60) | 102 (64) | 0.7 |
| Laboratory values during to the storm, median (IQR) |  |  |  |
| CRP (mg/L) | 48 (77) | 9 (17) | 0.08 |
| Proteinuria (g/dL) | 11 (12) | 0.85 (2.3) | ***0.02**** |
| Current laboratory values, median (IQR) |  |  |  |
| CRP (mg/L) | 2.7 (5.2) | 2.7 (7.3) | 0.7 |
| Proteinuria (g/day) | 3.1 (23) | 0 (1.9) | ***0.04**** |
| Creatinine (mg/dL) | 1.5 (3) | 1.2 (0.9) | 0.2 |
| Two exon 10 MEFV variant, n=108 (%) | 3 (100) | 94 (87) | 0.5 |
| M694V homozygous, n=3 (%) | 3 (100) | 73 (68) | 0.2 |
| ESRD development (overall), n (%) | 6 (67) | 71 (46) | 0.3 |
| Duration of b-DMARD (months), median (IQR) | 45 (104) | 62.5 (46.3) | 0.6 |
| Mortality, n, (%) | 3 (33) | 16 (10) | 0.07 |

*FMF: Familial Mediterranean fever, IQR: Interquartile range, CI: Confidence interval, SD: Standard deviation, e-GFR: Estimated glomerular filtration rate, CRP: C-reactive protein, CRF: Chronic renal failure, ESRD: End stage renal disease, b-DMARD: Biological disease modifying anti-rheumatic drug*

**^*^**Mann Whitney U test, **^Ɨ^** Fischer’s exact test

**Supplementary Figure S1**: Comparison of survival rate according to amyloid burden between G3 and G1 groups in patients with FMF-AA-a (Log-Rank p=0.007)


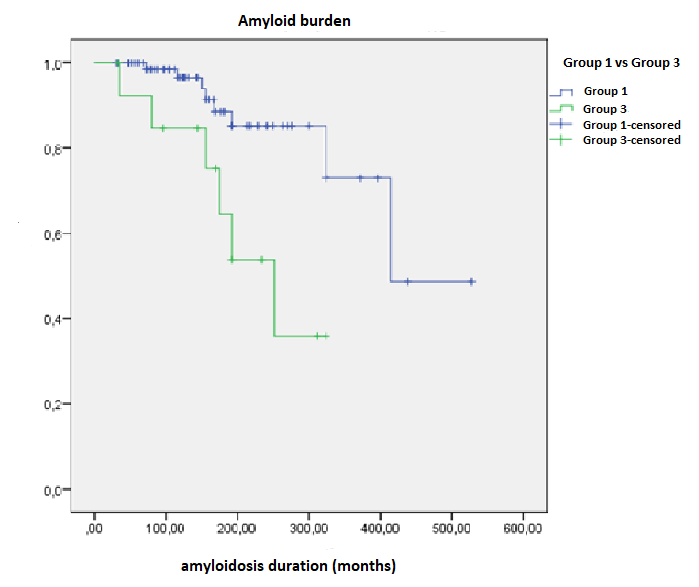


**Supplementary Figure S2:** Survival rate according to gender in patients with AA-a (Log Rank p=0.024)


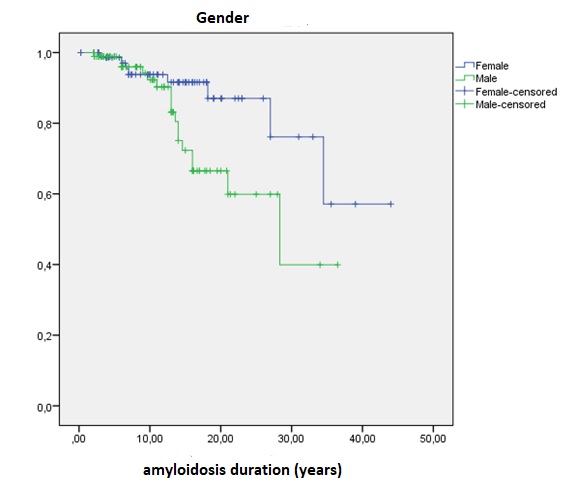


**Supplementary Figure S3:** Survival rate according to development of end-stage renal disease in patients with AA-a (Log-Rank p=0.019)


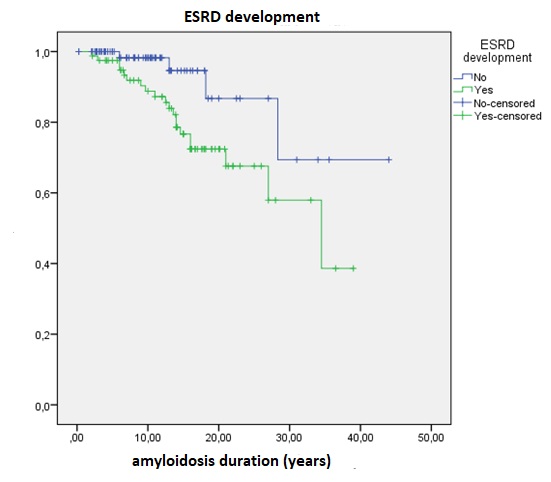


**Supplementary Figure S4**: Survival analysis according to the MEFV gene exon 10 variant status in patients with FMF (Log-rank p=0.028)


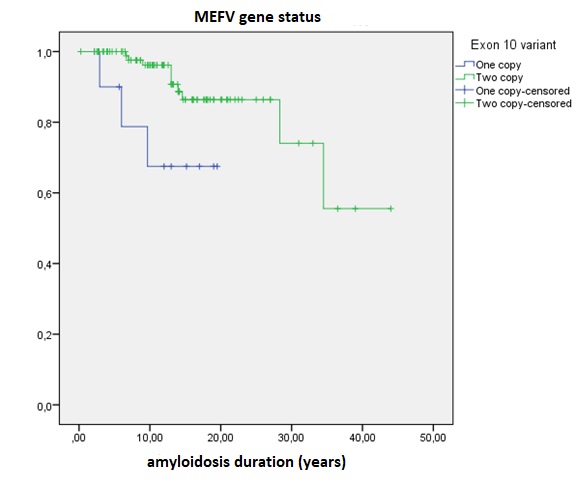


**Supplementary Figure S5:** Survival analysis according to MEFV gene exon 10 variant type in patients with FMF (Log-rank p=0.087)


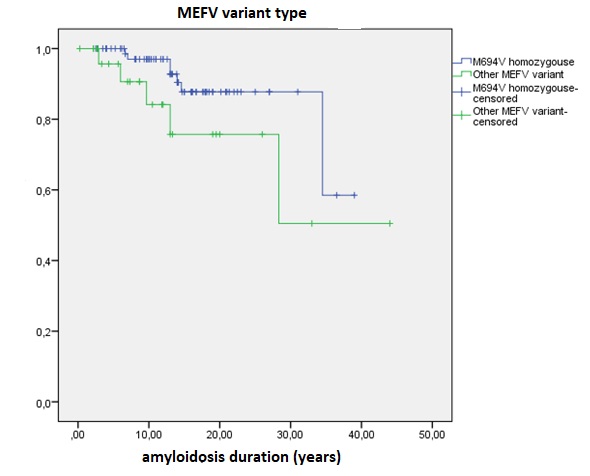


**Supplementary Figure S6:** Comparison of survival rate between patients with and without amyloid storm in patients with AA-a (Log-rank p=0.038)


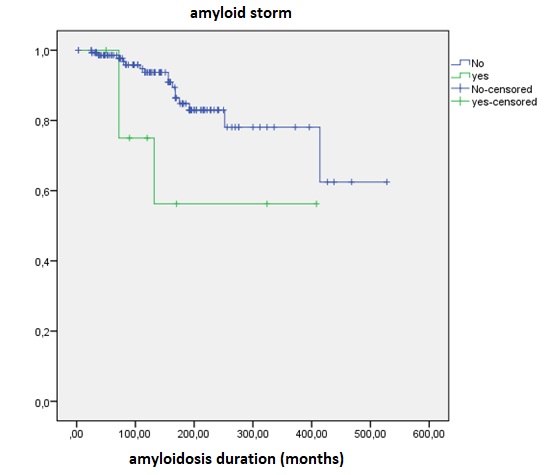

Supplement: kead465_Supplementary_Data [file kead465_supplementary_data.docx]
